# Case Report: Effective management of acute corneal hydrops with concurrent nystagmus and retinitis pigmentosa: combination of deep lamellar corneal suturing and anterior chamber gas injection

**DOI:** 10.3389/fmed.2026.1801611

**Published:** 2026-05-20

**Authors:** Ying Zhou, Abudusataer Aishan, YanChuan Yang, Xia Li

**Affiliations:** Department of Ophthalmology, Xinjiang 474 Hospital, Urumqi, Xinjiang, China

**Keywords:** acute corneal hydrops, deep lamellar corneal suturing, nystagmus, retinitis pigmentosa, therapy

## Abstract

**Background:**

Summarizing the clinical management and prognosis of acute corneal hydrops complicated by nystagmus and retinitis pigmentosa, this study provides a reference for the diagnosis and treatment of similar rare cases.

**Case presentation:**

Retrospective collection of data on three adolescent patients admitted to our hospital between December 2024 and October 2025 due to photophobia, eye pain, and tearing. Diagnosed with bilateral keratoconus concurrently with retinitis pigmentosa and nystagmus based on slit-lamp examination, corneal optical coherence tomography, macular optical coherence tomography, corneal topography, and fundus photography. Three patients presented with acute corneal edema due to rapid progression of keratoconus. Upon admission, they underwent deep lamellar corneal suturing in the edematous area combined with anterior chamber gas injection. Postoperative observations included the duration of corneal edema resolution, posterior lamellar closure, and corneal scar formation. The study analyzed the association among these three conditions and explored diagnostic and treatment approaches for keratoconus, nystagmus, and retinitis pigmentosa co-morbidity, along with specific implementation protocols and retinitis pigmentosa. In all three patients, postoperative corneal edema resolved promptly, with subsequent successful healing of the posterior elastic layer. No complications such as corneal perforation or neovascularization occurred. Follow-up examinations 1 to 3 months post-surgery revealed significant improvement in the patient’s photophobia and ocular pain symptoms, with only minimal corneal opacities or hazes remaining. Quality of life showed marked enhancement.

**Conclusion:**

Deep lamellar corneal suturing combined with anterior chamber gas injection safely and effectively controls acute corneal edema in patients with keratoconus complicated by retinitis pigmentosa and nystagmus. This approach effectively prevents severe complications such as corneal perforation and neovascularization, making it the preferred clinical solution for improving the prognosis of such low-vision patients with acute corneal hydrops.

## 1 Introduction

Keratoconus (KC) is a common bilateral corneal ectasia characterized by central or paracentral corneal thinning and steepening. If left untreated, it may lead to severe visual impairment. Its global prevalence ranges from 0.05 to 0.23% (1), its primary risk factors include rubbing the eyes and genetic predisposition. The condition often leads to high irregular astigmatism, with a high susceptibility to acute corneal edema in advanced stages. Following resolution of the edema, scarring frequently persists, causing severe visual impairment. Treatment plans should be tailored to the individual’s condition. In the early stages, vision can be corrected using eyeglasses or contact lenses. If the condition progresses or corrective measures prove ineffective, surgical options such as corneal collagen cross-linking or corneal transplantation may be considered (2). Clinically, acute corneal edema caused by rupture of the posterior elastic layer is termed “hydrops.” For such patients, corneal suturing is an effective method to prevent corneal perforation, significantly reduce corneal edema, and provide an opportunity for a second-stage lamellar corneal transplant (3).

The onset of keratoconus is often associated with systemic and ocular diseases, systemic conditions such as atopic diseases, Down syndrome, and connective tissue disorders increase the risk of developing keratoconus. Isolated ocular disorders such as Leber congenital amaurosis (LCA), retinitis pigmentosa (RP), and retinopathy of prematurity may also coexist with keratoconus (4). Retinitis pigmentosa is a group of hereditary retinal pigmentary dystrophies characterized by night blindness as the initial symptom. Vision progressively deteriorates due to photoreceptor cell apoptosis and retinal pigment epithelium atrophy. Typical fundus findings include a waxy yellow atrophy of the optic disc, thinning of retinal arterioles, and peripheral retinal bone-spike-like pigment deposits (5). Currently, there are few reported cases of coexisting retinitis pigmentosa with keratoconus and nystagmus. The relationship between these three conditions and their underlying pathogenesis remain unclear. Whether retinitis pigmentosa serves as a triggering factor for keratoconus requires further clinical data to support.

This paper reports three cases of patients presenting with concurrent nystagmus, retinitis pigmentosa, and keratoconus, all of whom sought medical attention due to pronounced symptoms triggered by acute corneal edema. Patients with this condition have inherently poor visual function due to underlying eye diseases, often failing to detect vision changes associated with keratoconus in its early stages. Diagnosis typically occurs only when acute corneal edema causes significant ocular irritation symptoms and vision loss. This study aims to share the clinical characteristics, surgical treatment experience, and prognosis of this rare case category, while emphasizing that when encountering patients with retinitis pigmentosa accompanied by nystagmus in clinical practice, attention must be paid to corneal-related examinations to avoid overlooking corneal pathology.

## 2 Case presentation

### 2.1 Case 1 presentation

**Figure 1:**
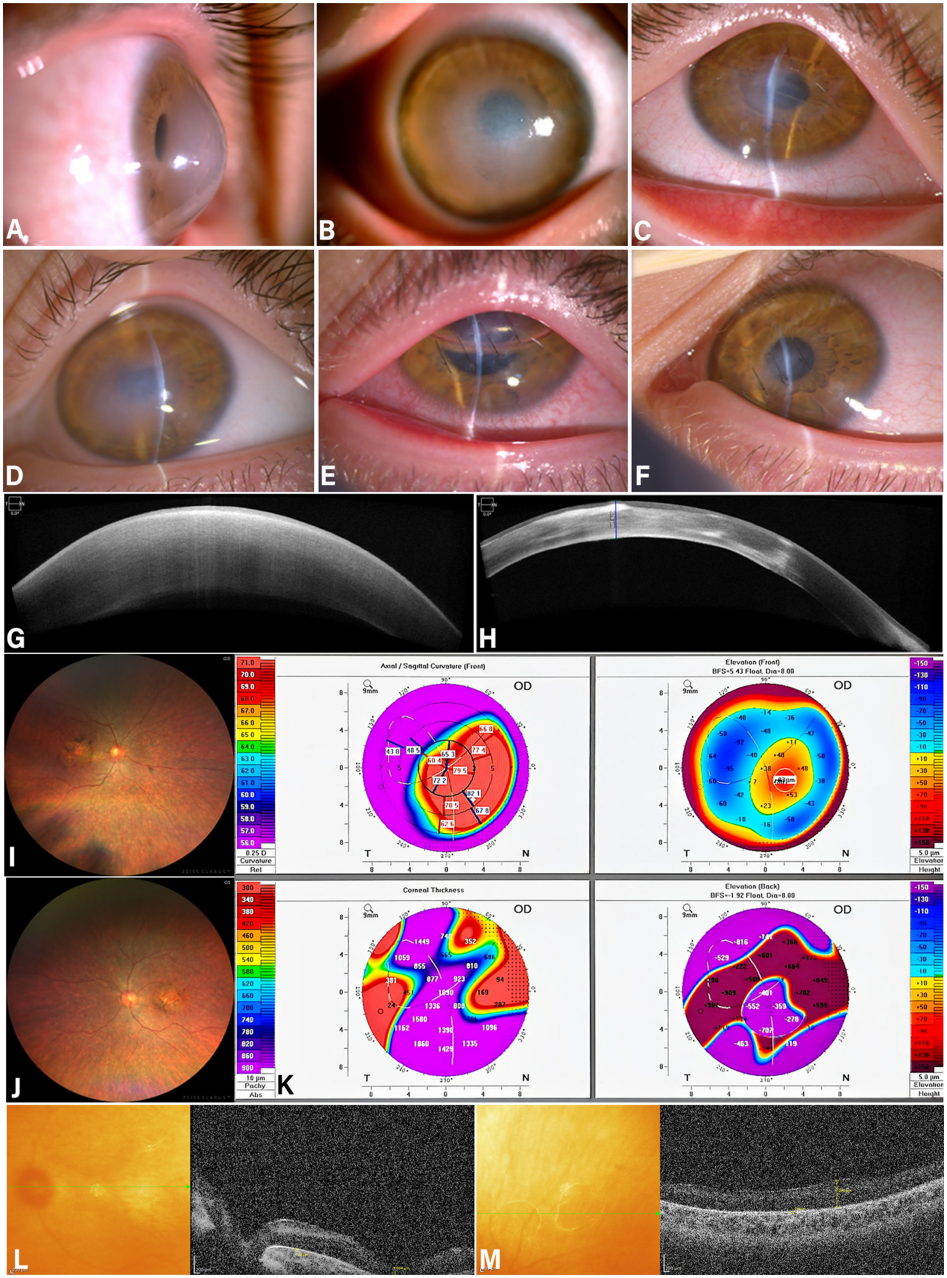
**(A, B)** The right eye with acute corneal edema, protrudes forward in a conical shape, Musson’s sign and Vogt’s striae observed. **(C)** Corneal edema resolved following deep corneal suturing combined with anterior chamber gas injection, residual corneal opacity. **(D–F)** Three months after right eye surgery, the left eye developed acute corneal edema. Undergo the same surgery, the left corneal edema resolved within 3 days postoperatively **(E)**. At 2 months postoperatively, the left eye showed superior prognosis compared to the right eye, with milder corneal scarring. Previous Section: Optical Coherence Tomography **(G)** show that right corneal prolapse, subepithelial fluid accumulation, central corneal edema with thickening, posterior lamellar membrane rupture. **(H)** Two months postoperatively, corneal healing was satisfactory with residual patchy corneal haze. Fundus Photography **(I, J)** show macular atrophy with peppercorn-like pigmentation. Optical Coherence Tomography of the Macula **(L, M)** show that the fovea centralis of both eyes shows deepening and enlargement, with disappearance of the retinal ellipsoid zone, atrophy of the retinal pigment epithelium (RPE) layer, and pigmentary degeneration. Corneal topography scan: **(K)** Maximum K-value of the right eye: 83.0D Thinnest point corneal thickness: 24μm Anterior surface height: 87μm Posterior surface height: 962μm.

A 10-year-old male patient was admitted to the hospital presenting with decreased vision in the right eye, eye pain, and photophobia for one month. The patient has exhibited nystagmus since infancy. The ophthalmic examination findings are as follows: horizontal nystagmus in both eyes; corrected visual acuity is light perception in both eyes, with inaccurate light localization; Right intraocular pressure: 15 mmHg; left intraocular pressure: 14 mmHg. Right eye conjunctiva shows mixed hyperemia. The cornea exhibits a conical protrusion forward, with stromal edema accompanied by bullous changes, Posterior elastic layer shows folds, observed positive Musson’s sign and Vogt’s striae (Figures 1A,B). Specialized eye examination: anterior segment optical coherence tomography (OCT) reveals: right eye corneal protrusion, subepithelial fluid accumulation, central corneal edema with thickening, posterior lamellar membrane rupture (Figure 1G). Corneal topography show the right eye maximum K-value 83.0D, thinnest point corneal thickness 24 μm, anterior surface height 87 μm, posterior surface height 962 μm (Figure 1K), fundus photography reveals bilateral macular atrophy with peppercorn-like pigment deposits (Figures 1I,J). Optical coherence tomography (OCT) of the posterior segment reveals bilateral deepening and enlargement of the macular fovea, disappearance of the retinal ellipsoid zone, atrophy of the retinal pigment epithelium (RPE) layer, and pigmentary degeneration (Figures 1L,M). Diagnosis: the right eye: acute corneal hydrops; the left eye: Keratoconus (early stage) Both eyes: Nystagmus, Retinitis pigmentosa. Course of Treatment: Following emergency admission, the patient underwent comprehensive examinations. Under general anesthesia, a combined procedure of deep lamellar corneal suturing and anterior chamber gas injection was performed on the right eye. During surgery, the elastic layer tear was first localized, then interrupted sutures were placed perpendicular to the tear to close the deep lamellar corneal layer.

#### 2.1.1 Postoperative instructions

Maintain supine position for 24 h. Release small amounts of gas 4 h postoperatively to prevent pupillary block. On the first postoperative day, corneal edema subsided and intraocular pressure normalized. At the one-month follow-up, light perception was restored, light localization was accurate, intraocular pressure remained normal, corneal edema had significantly resolved, and symptoms of photophobia and ocular pain had alleviated. Two months postoperatively, visual acuity is light perception, light projection is normal, intraocular pressure, and corneal healing were satisfactory. Sutures were removed, and no corneal neovascularization but residual lamellar corneal opacity was detected (Figures 1C,H). Three months postoperatively, the left eye developed acute corneal edema (Figure 1D), following a thorough examination, the patient underwent deep lamellar keratoplasty combined with anterior chamber gas injection in the left eye. Corneal edema resolved by the third postoperative day (Figure 1E). At the 2-month postoperative follow-up, the left eye showed a better prognosis than the right eye, with less corneal scarring compared to the right eye (Figure 1F), consideration of the duration and severity of preoperative edema. Due to the patient’s limited financial resources and the presence of bilateral nystagmus and retinitis pigmentosa, which resulted in poor visual development, a corneal transplant was not performed. However, this surgery effectively alleviated the patient’s discomfort and improved their quality of life.

### 2.2 Case 2 presentation

**Figure 2:**
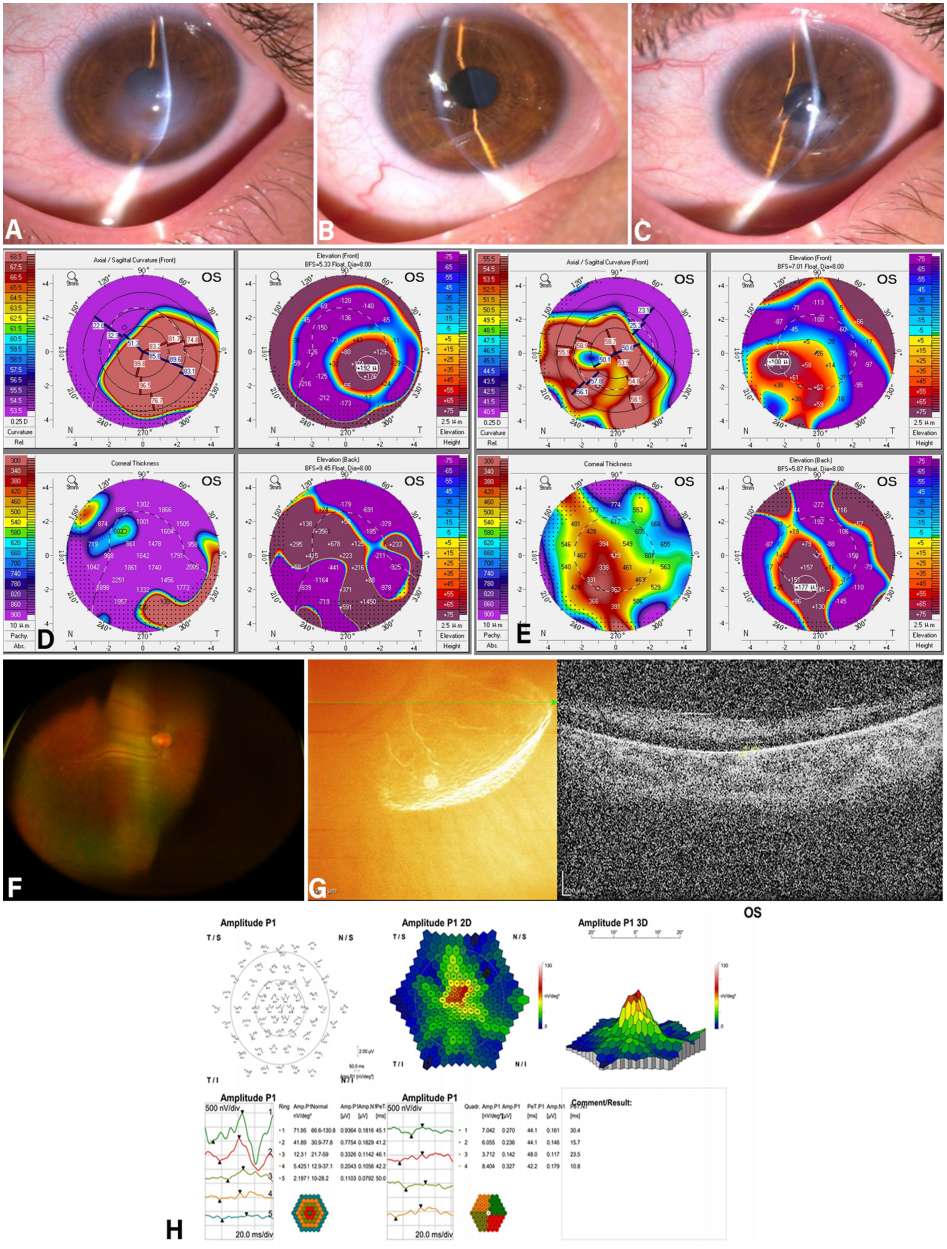
Preoperative examination: Slit-lamp examination indicate corneal edema in the right eye **(A)**, and anterior segment OCT shows corneal hydrops with stromal clefts **(B)**. The corneal topography demonstrates features consistent with keratoconus: Right eye maximum K-value: 100.4D Corneal edema thickness: 1372 μm Anterior surface height: 91μm Posterior surface height: 439μm **(E)**. Three months postoperatively, corneal edema in the right eye has completely resolved **(C, D)**. Corneal topography: Right eye maximum K-value 67.3D, corneal edema thickness 451μm, anterior surface height 53μm, posterior surface height 136 μm **(F)**. Fundus photography reveals a waxy yellow disc in both eyes, with macular retinal atrophy accompanied by pigment encirclement **(G)**. Macular OCT reveals flattening of the foveal center and atrophy of the retinal pigment epithelium layer **(H)**.

A 17-year-old male presented to the emergency department with complaints of left eye pain, redness, and decreased vision for 3 days. Past medical history: Poor eyesight since childhood. Ophthalmic examination: Horizontal nystagmus in both eyes. The best corrected visual acuity in right eye: manual / 10 cm. The best corrected visual acuity in left eye: light perception only. Light localization is inaccurate, the right eye intraocular pressure: 16 mmHg Left eye intraocular pressure: 18 mmHg. Slit-lamp examination reveals: Left eye shows conjunctival hyperemia and corneal edema, with a faint central posterior corneal elastic layer rupture visible. Right eye shows no conjunctival hyperemia, with the cornea presenting a conical protrusion (Figures 2A,B). On Pentacam, the maximum K-value: 102.0D, the thinnest pachymetry was 571 μm, the maximum elecation was +192 on the anterior elevation map and +415 on the posterior elecation maps in the left eye (Figure 2D). Due to the patient’s pronounced nystagmus and severe photophobia, clear corneal OCT images could not be obtained. Further fundus photography revealed diffuse osteoblast-like pigment deposition in the peripheral retina of the right eye (Figure 2F). Optical coherence tomography of the macula revealed disappearance of the retinal ellipsoid zone and atrophy of the retinal pigment epithelium layer (Figure 2G). Diagnosis: Acute corneal hydrops in the left eye, keratoconus in the right eye (end-stage), bilateral nystagmus, bilateral retinitis pigmentosa. Following admission, the patient underwent deep lamellar corneal suturing combined with anterior chamber gas injection in the left eye using the same technique as previously described. Postoperatively, corneal edema gradually subsided, symptoms improved, and recovery proceeded smoothly. Due to academic commitments, the patient did not attend regular follow-up appointments. At the 4-month postoperative follow-up, slit-lamp examination revealed mild conjunctival hyperemia in the left eye, resolution of corneal edema, suture fixation without displacement, and residual patchy opacities in the central cornea (Figure 2C). On Pentacam, the maximum K-value: 63.2D, the thinnest pachymetry was 306 μm, the maximum elecation was +108 on the anterior elevation map and +177 on the posterior elecation maps in the left eye (Figure 2E). Postoperative electroretinogram findings: Central 1–2 rings show mild reduction in amplitude density; 3–5 rings show severe reduction in amplitude density; 3 dimension spike shows moderate reduction; all quadrants show severe reduction in amplitude density (Figure 2H).

### 2.3 Case 3 presentation

**Figure 3:**
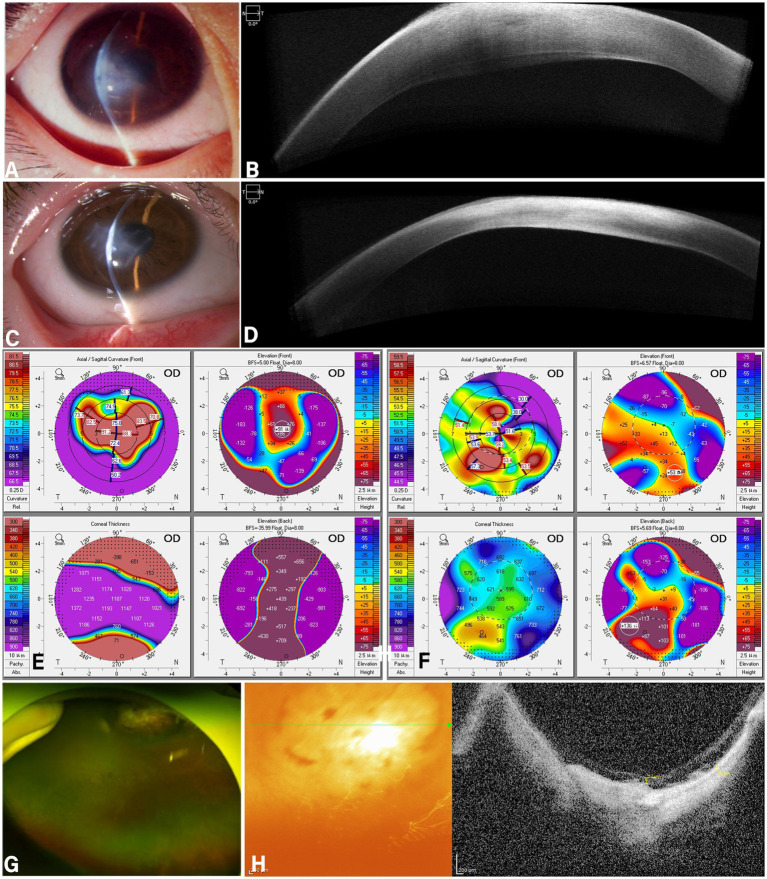
Slit lamp examination **(A)** Left eye: Conjunctival hyperemia, corneal edema, with a faintly visible rupture of the central corneal posterior elastic layer. **(B)** Right eye: Corneal transparency, corneal cone-shaped protrusion. **(C)** Follow-up examination at 4 months postoperatively revealed mild conjunctival hyperemia in the left eye, resolution of corneal edema, suture fixation without displacement, and residual patchy opacities in the central cornea. Corneal topography **(D)** shows the left eye with a maximum K-value of 102.0D, thinnest point thickness of 571μm, anterior surface height of 192μm, and posterior surface height of 415μm. **(E)** Four months post-surgery, corneal topography revealed a maximum K-value of 63.2D in the left eye, with a thinnest point thickness of 306 μm, anterior surface height of 108 μm, and posterior surface height of 177 μm. **(F)** Fundus photography reveals diffuse osteoclast-like pigment deposition in the peripheral retina of the right eye. **(G)** Macular OCT reveals disappearance of the retinal ellipsoid zone and atrophy of the retinal pigment epithelium (RPE) layer. **(H)** Electroretinogram findings: Central 1-2 rings show mild reduction in amplitude density, rings 3-5 show severe reduction, and the 3D spike shows moderate reduction; all quadrants in the quadrant diagram show severe reduction in amplitude density.

A 12-year-old male patient was admitted for photophobia, eye pain, and decreased vision in the right eye for 7 days, on admission examination: horizontal nystagmus in both eyes; the best corrected visual acuity in the right eye: manual at 30 cm; the best corrected visual acuity in the left eye: 6/60. Slit-lamp examination and anterior segment OCT both revealed corneal edema in the right eye (Figures 3A,B). On Pentacam, the maximum K-value: 100.4D, the corneal edema thickness: 1372 μm, the maximum elecation was +91 on the anterior elevation map and +439 on the posterior elecation maps in the right eye (Figure 3E). Fundus photography reveals a waxy yellow disc in both eyes, with macular retinal atrophy accompanied by pigment encirclement (Figure 3G). Optical coherence tomography of the macula demonstrates flattening of the foveal center, atrophy of the retinal pigment epithelium layer (Figure 3H), diagnosed that acute corneal hydrops in the right eye, bilateral retinitis pigmentosa, and bilateral nystagmus. Following admission, the patient underwent right eye deep lamellar keratoplasty combined with anterior chamber gas injection.

The first day after surgery the right eye visual acuity: hand motion at 30 cm, intraocular pressure normal corneal edema reduced eye pain and photophobia symptoms relieved discharged. A slit-lamp examination three months postoperatively revealed that the corneal edema in the right eye has completely resolved, leaving behind a corneal opacity (Figures 3C,D). On Pentacam, the maximum K-value: 67.3D, the corneal edema thickness: 451 μm, the maximum elecation was +53 on the anterior elevation map and +136 on the posterior elecation maps in the right eye (Figure 3F).

## 3 Discussion

The three patients included in this study were all special cases—simultaneously presenting with nystagmus, retinitis pigmentosa, and keratoconus, with keratoconus acutely onset during adolescence. Eye pain, photophobia, and subjective deterioration in vision were their initial presenting symptoms. These three patients share a common characteristic: due to poor baseline visual function caused by retinitis pigmentosa and nystagmus, they were unable to detect visual changes caused by keratoconus at an early stage. Consequently, none had received formal keratoconus treatment prior to seeking medical care. They only sought treatment after the condition progressed to acute corneal hydrops, resulting in significant vision loss and ocular irritation symptoms. This phenomenon suggests that corneal pathologies are prone to being overlooked in patients with underlying eye conditions such as retinitis pigmentosa and nystagmus, which are associated with low vision. Clinicians should exercise heightened vigilance when evaluating such patients.

The pathogenesis of keratoconus remains incompletely understood, involving multiple factors including genetics, immunity, biomechanics, stromal cells, oxidative stress, environment, and behavior. Among these, genetic factors represent one of the key contributing causes (6). Research has found that the inheritance patterns of keratoconus include autosomal dominant inheritance, autosomal recessive inheritance, or sporadic inheritance. Genome-wide association studies have identified multiple genetic loci associated with keratoconus, including LOX, FOXO1, HGF, and FNDC3B (7). The rs3735520 variant located upstream of the hepatocyte growth factor gene is associated with keratoconus in American and Australian populations (8). Li et al. demonstrates that SLC4A11, TGFBI, PIKFYVE, and ZEB1 are associated with keratoconus (9). Li et al. performed triplet whole-exome sequencing on 200 sporadic keratoconus patients in China and their unaffected parents, identifying the IMPDH1 gene. Based on guidelines from the American College of Medical Genetics and Genomics, the c. T464C variant in the IMPDH1 gene was defined as likely pathogenic (10). The above studies not only continuously expand the genetic landscape of keratoconus, highlighting the disease’s genetic heterogeneity, but also provide crucial clues for future functional validation. Besides genetic factors, eye rubbing and exposure to sunlight may cause oxidative damage to the cornea in keratoconus, because the amount of enzymes in the cornea responsible for handling reactive oxygen species (ROS), such as aldehyde dehydrogenase 3 (ALDH3) and superoxide dismutase, decreases, which leads to the degenerative process of corneal thinning and vision loss (11).

An increasing number of researchers have noted that keratoconus may coexist with various systemic disorders (such as Down syndrome) and ocular conditions (including retinitis pigmentosa, Leber congenital amaurosis, retinopathy of prematurity, Fuchs’ corneal dystrophy, and allergic conjunctivitis) (4). Among these, retinitis pigmentosa is a type of hereditary retinal dystrophy with a global prevalence of 1 in 7,000 to 1 in 3,000, and approximately 1 in 4,000 in China, its inheritance patterns are primarily autosomal recessive, autosomal dominant, and X-linked recessive (5). In this study, Case 1 is a child born to consanguineous parents (cousins who married). The younger brother of Case 2, who is three years younger, was also diagnosed at our hospital with bilateral nystagmus and bilateral keratoconus. No other family members have a history of related diseases, consistent with the characteristics of autosomal recessive inheritance. Regarding the coexistence mechanism of nystagmus, retinitis pigmentosa, and keratoconus, no definitive conclusions have been reached at present. Zeki Fikret et al. submitted that patients with retinitis pigmentosa exhibit elevated levels of pro-inflammatory cytokines and increased oxidative stress in the vitreous cavity, which not only damages the retina but may also affect the cornea (12). Oxidative stress and immune responses are also significant pathogenic factors in keratoconus (6). In retinal degenerative diseases, activated microglia and macrophages release cytokines such as the IL-1 family, promoting photoreceptor apoptosis (13). In patients with keratoconus, pro-inflammatory factors such as IL-1, IL-6, and TNF-*α* are also highly expressed in corneal tissue and tears. These factors lead to degradation of the corneal extracellular matrix and corneal thinning (4). Some scholars have performed a proteomic analysis of the tears with keratoconus patients and the relation between keratoconus severity and the tear proteins. They have demonstrated that the protein composition of tears is changed in keratoconus by increased levels of proteins with inflammatory properties such as albumin or by decreased levels of pro teins with anti-inflammatory properties such as lactoferrin. Lysozyme is positively correlated with the maximum corneal curvature, while lactoferrin is negatively correlated with corneal curvature. This indicates that the higher the local inflammation and the lower the anti-inflammatory ability in the tears of keratoconus patients, the more severe the disease (11). Based on this, it is speculated that inflammatory responses and oxidative stress may represent the common pathological basis underlying the coexistence of these three diseases. Furthermore, genetic testing may reveal the molecular mechanisms underlying the association among these three factors. However, due to objective constraints, the patients in this study did not undergo the relevant testing, leaving this hypothesis to be verified in subsequent research.

In this study, three patients with retinitis pigmentosa require careful differentiation from Leber congenital amaurosis. Leber congenital amaurosis is a rare group of inherited retinal disorders characterized by early onset and severe visual impairment. Currently, 27 relevant genes have been identified, exhibiting significant genetic and clinical heterogeneity (14). Certain subtypes of Leber congenital amaurosis (such as mutations in the CRB1 and RDH12 genes) may present with nystagmus, retinal pigment deposits, and the finger-eye sign, exhibiting clinical manifestations similar to those observed in the cases studied herein (14). Due to objective constraints such as nystagmus and financial limitations, patients in this study were unable to undergo comprehensive electroretinography, visual field testing, and genetic testing, making it difficult to differentiate between the two conditions. However, given that Leber congenital amaurosis manifests earlier than retinitis pigmentosa and causes more severe visual impairment, combined with the progression of visual changes in these patients, clinicians in this study were more inclined to clinically diagnose the three patients with retinitis pigmentosa.

Acute corneal edema is a rare complication of keratoconus. This condition is primarily caused by spontaneous rupture of the posterior elastic layer (Descemet’s membrane, DM), allowing aqueous humor to infiltrate the corneal stroma, severely impairing patients’ vision and quality of life. According to reports, the incidence of acute corneal edema in patients with keratoconus ranges from 2.4 to 3%, while in patients with clear marginal corneal dystrophy, it ranges from 6 to 11.5% (15). However, reports of progressive corneal ectasia following refractive corneal surgery and ectasia-induced acute corneal edema after corneal transplantation are relatively rare. Risk factors associated with acute corneal edema include early-onset keratoconus, contact lens-related microtrauma, eye rubbing, allergic conjunctivitis, and Down syndrome (16).

In clinical practice, conservative management of acute corneal edema primarily involves hypertonic eye drops, antibiotics, cycloplegic agents, and corticosteroids. However, the healing process is prolonged (typically 2–6 months), and edema resolution often leaves residual scarring. Furthermore, prolonged inflammation and edema may promote corneal neovascularization, increasing the difficulty of subsequent corneal transplantation and the risk of graft rejection. To accelerate the resolution of edema and improve prognosis, various surgical approaches have been explored in clinical practice. Sayadi et al. improved corneal edema by injecting sulfur hexafluoride (SF6) into the anterior chamber. Although edema resolved after one month, risks include corneal endothelial damage, pupillary block leading to secondary glaucoma, and the need for multiple injections (17). Basu et al. proposed that the key to resolving corneal edema lies in the reattachment of the posterior lamella to the posterior corneal stroma and the migration of endothelial cells to repair the tear. Simple anterior chamber gas injection is insufficient to close larger posterior lamellar tears (18). Zhao et al. reported that pressurized suturing combined with anterior chamber air injection and thermal keratoplasty achieved favorable outcomes in the treatment of acute corneal edema (19).

It should be noted that previous reports have primarily focused on the treatment of acute corneal edema in isolated keratoconus. However, there are relatively few reports concerning cases complicated by retinitis pigmentosa and nystagmus. Patients in this study, who also suffered from retinitis pigmentosa, macular degeneration, and nystagmus, experienced only limited visual improvement even after corneal transplantation. Additionally, the patient’s poor vision makes it difficult to detect complications such as wound leakage, suture issues, or graft rejection early on after surgery. Therefore, corneal transplantation was not selected.

We ultimately opted for deep lamellar keratoplasty combined with anterior chamber gas injection. This procedure directly closes posterior lamellar tears, promotes corneal endothelial repair, accelerates edema resolution, and effectively prevents severe complications such as corneal perforation and neovascularization. Simultaneously, it significantly alleviates patient discomfort including photophobia and ocular pain, aligning with the core therapeutic objectives of “preserving the eye and improving quality of life” for such patients. Follow-up results showed that all three patients recovered well postoperatively, with only minimal corneal scarring remaining, further confirming the efficacy of this surgical technique.

## 4 Conclusion

For patients with acute corneal hydrops complicated by retinitis pigmentosa and nystagmus, deep lamellar keratoplasty combined with anterior chamber gas injection represents a safe and effective treatment option. Apidly resolves corneal edema, promotes healing of the posterior elastic layer, prevents serious complications such as corneal perforation, and improves patients’ quality of life. However, this study has limitations including a small sample size and a short follow-up period. Future research should extend the follow-up duration to continuously monitor long-term corneal changes in patients. Another limitation is that three patients refused to undergo genetic testing, therefore can not provide additional evidence for exploring the mechanisms of disease comorbidity. Additionally, we did not evaluate the status of endothelium cells before or after the operation. In future studies, we should focus on the status of corneal endothelial cells to further validate the safety of the surgery. Furthermore, when clinically examining patients with retinitis pigmentosa, attention should not be limited to retinal lesions alone. A thorough examination of the cornea is also essential to screen for potential corneal pathologies. This enables early identification, personalized treatment, and enhanced patient education, thereby maximizing the preservation of visual function and ensuring quality of life.

## Data Availability

The original contributions presented in the study are included in the article/Supplementary material, further inquiries can be directed to the corresponding author.

## References

[1] Bayat K Pooyan P Feizi S Ahmadieh H Hafezi F Pourhoseingholi MA . Structural alterations in the retina and choroid of keratoconus patients detected by optical coherence tomography: a systematic review and meta-analysis. Surv Ophthalmol. (2025) 71:892–908. doi: 10.1016/j.survophthal.2025.11.004, 41197878

[2] Son HS Friedrich M Jun AS Soiberman US. Advancements and innovations in Keratoconus management: a review of current practices. J Clin Med. (2025) 14:7491. doi: 10.3390/jcm14217491, 41226888 PMC12609086

[3] Kaur M Balaji A Titiyal JS Bansal M Raj R Namdev V. Intraoperative optical coherence tomography-guided compression sutures in acute corneal hydrops—surgical technique and review of literature. Indian J Ophthalmol. (2025) 73:1779–85. doi: 10.4103/IJO.IJO_2795_24, 40971506 PMC12707409

[4] Navel V Malecaze J Pereira B Baker JS Malecaze F Sapin V . Oxidative and antioxidative stress markers in keratoconus: a systematic review and meta-analysis. Acta Ophthalmol. (2021) 99:e777–94. doi: 10.1111/aos.14714, 33354927

[5] Liu W Liu S Li P Yao K. Retinitis Pigmentosa: Progress in molecular pathology and Biotherapeutical strategies. Int J Mol Sci. (2022) 23:4883. doi: 10.3390/ijms23094883, 35563274 PMC9101511

[6] Singh RB Koh S Sharma N Woreta FA Hafezi F Dua HS . Keratoconus. Nat Rev Dis Primers. (2024) 10:81. doi: 10.1038/s41572-024-00565-3, 39448666

[7] Sahebjada S Schache M Richardson AJ Snibson G Daniell M Baird PN. Association of the hepatocyte growth factor gene with keratoconus in an Australian population. PLoS One. (2014) 9:e84067. doi: 10.1371/journal.pone.0084067, 24416191 PMC3885514

[8] Cheng WY Yang SY Huang XY Zi FY Li HP Sheng XL. Identification of genetic variants in five chinese families with keratoconus: pathogenicity analysis and characteristics of parental corneal topography. Front Genet. (2022) 13:978684. doi: 10.3389/fgene.2022.978684, 36276932 PMC9583916

[9] Li X Yao Y Xing S Zheng YH Zhou Y Yu X . Trio-based whole-exome sequencing of 200 Chinese patients with keratoconus. Exp Eye Res. (2024) 248:110109. doi: 10.1016/j.exer.2024.110109, 39326774

[10] Zemba M Zaharia AC Dumitrescu OM. Association of retinitis pigmentosa and advanced keratoconus in siblings. Rom J Ophthalmol. (2020) 64:313–20. doi: 10.22336/rjo.2020.52, 33367168 PMC7739560

[11] Burcel MG Constantin M Ionita G Dana D Catalina L Dan S . Levels of lactoferrin, lysozyme and albumin in the tear film of keratoconus patients and their correlations with important parameters of the disease. Rev Romana Med Lab. (2020) 2:153–61. doi: 10.2478/RRLM-2020-0018

[12] Zeki Fikret C Ucgun NI Karaca EE Evren Kemer O. Corneal characteristics in patients with retinitis pigmentosa. Photodiagn Photodyn Ther. (2023) 42:103554. doi: 10.1016/j.pdpdt.2023.103554, 37030435

[13] Kaur G Singh NK. The role of inflammation in retinal neurodegeneration and degenerative diseases. Int J Mol Sci. (2021) 23:386. doi: 10.3390/ijms23010386, 35008812 PMC8745623

[14] Chinese Hereditary Ocular Disease Diagnosis and Treatment Group, Chinese Hereditary Ocular Disease Alliance. Chinese expert consensus on diagnosis and treatment of Leber congenital amaurosis. Chin J Exp Ophthalmol. (2023) 41:833–42. doi: 10.3760/cma.j.cn115989-20230523-00188

[15] Bafna RK Kalra N Asif MI Beniwal A Lata S Sharma SV . Management of acute corneal hydrops—current perspectives. Indian J Ophthalmol. (2024) 72:495–507. doi: 10.4103/IJO.IJO_2160_23, 38317314 PMC11149508

[16] Singh M Prasad N Sinha BP. Management of acute corneal hydrops with compression sutures and air tamponade. Indian J Ophthalmol. (2022) 70:2210. doi: 10.4103/ijo.IJO_1258_22, 35648028 PMC9359272

[17] Sayadi JJ Lam H Lin CC Myung D. Management of acute corneal hydrops with intracameral gas injection. Am J Ophthalmol Case Rep. (2020) 20:100994. doi: 10.1016/j.ajoc.2020.100994, 33319122 PMC7726327

[18] Basu S Vaddavalli PK Vemuganti GK Ali MH Murthy SI. Anterior segment optical coherence tomography features of acute corneal hydrops. Cornea. (2012) 31:479–85. doi: 10.1097/ICO.0b013e318223988e, 22314821

[19] Zhao Z Wu S Ren W Zheng Q Ye C Kim AD . Compression sutures combined with intracameral air injection versus thermokeratoplasty for acute corneal hydrops: a prospective-randomised trial. Br J Ophthalmol. (2021) 105:1645–50. doi: 10.1136/bjophthalmol-2020-316414, 33011684

