# Supplementary material for: Case Report: Effective management of acute corneal hydrops with concurrent nystagmus and retinitis pigmentosa: combination of deep lamellar corneal suturing and anterior chamber gas injection
Source: Front Med (Lausanne). 2026 May 20;13:1801611. doi: 10.3389/fmed.2026.1801611 (PMC13229636; doi:10.3389/fmed.2026.1801611)
Supplement: Supplementary file 1 [file Data_Sheet_1.pdf]

Supplementary Material

1 Supplementary Data

Supplementary Material should be uploaded separately on submission. Please include any supplementary data, figures and/or tables.

Supplementary material is not typeset so please ensure that all information is clearly presented, the appropriate caption is included in the file and not in the manuscript, and that the style conforms to the rest of the article.

2 Supplementary Figures and Tables

For more information on Supplementary Material and for details on the different file types accepted, please see here.

2.1 Supplementary Figures

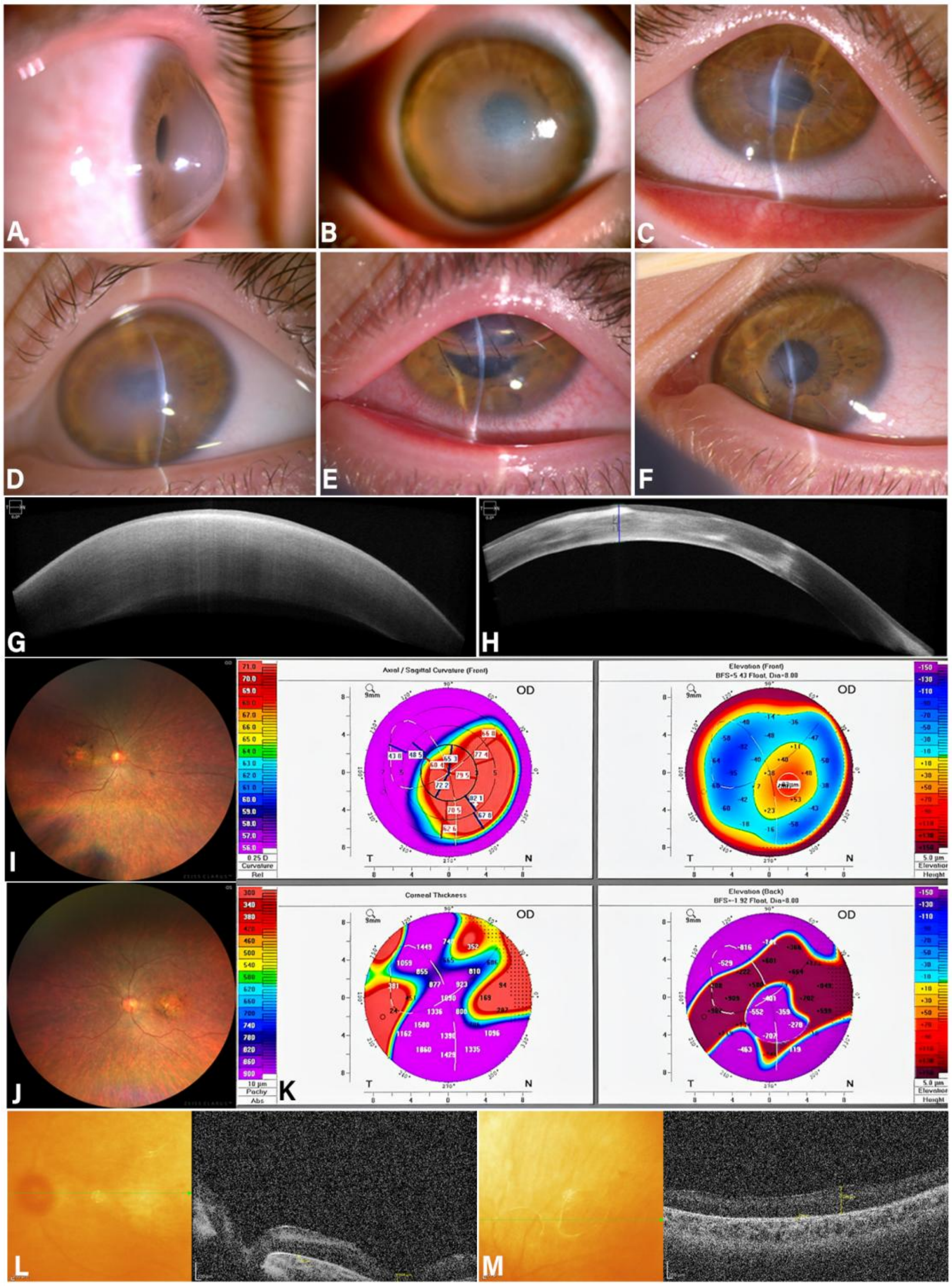

Supplementary Figure 1.

(A,B)The right eye with acute corneal edema,protrudes forward in a conical shape, Musson's sign and Vogt's striae observed.

(C) Corneal edema resolved following deep corneal suturing combined with anterior chamber gas injection,residual corneal opacity.

(D-F)Three months after right eye surgery, the left eye developed acute corneal edema.

(E) Undergo the same surgery,the left corneal edema resolved within 3 days postoperatively . At 2 months postoperatively, the left eye showed superior prognosis compared to the right eye, with milder corneal scarring.

(G)Optical Coherence Tomography show that right corneal prolapse, subepithelial fluid accumulation, central corneal edema with thickening, posterior lamellar membrane rupture.

(H) Two months postoperatively, corneal healing was satisfactory with residual patchy corneal haze.

(I,J) Fundus Photography show that macular atrophy with peppercorn-like pigmentation.

(K)Corneal topography scan:Maximum K-value of the right eye: 83.0D Thinnest point corneal thickness: 24μm Anterior surface height: 87μm Posterior surface height: 962μm.

(L,M)Optical Coherence Tomography of the Macula and Fundus Photography show that the fovea centralis of both eyes shows deepening and enlargement, with disappearance of the retinal ellipsoid zone, atrophy of the retinal pigment epithelium (RPE) layer, and pigmentary degeneration.Macular atrophy with peppercorn-like pigmentation.

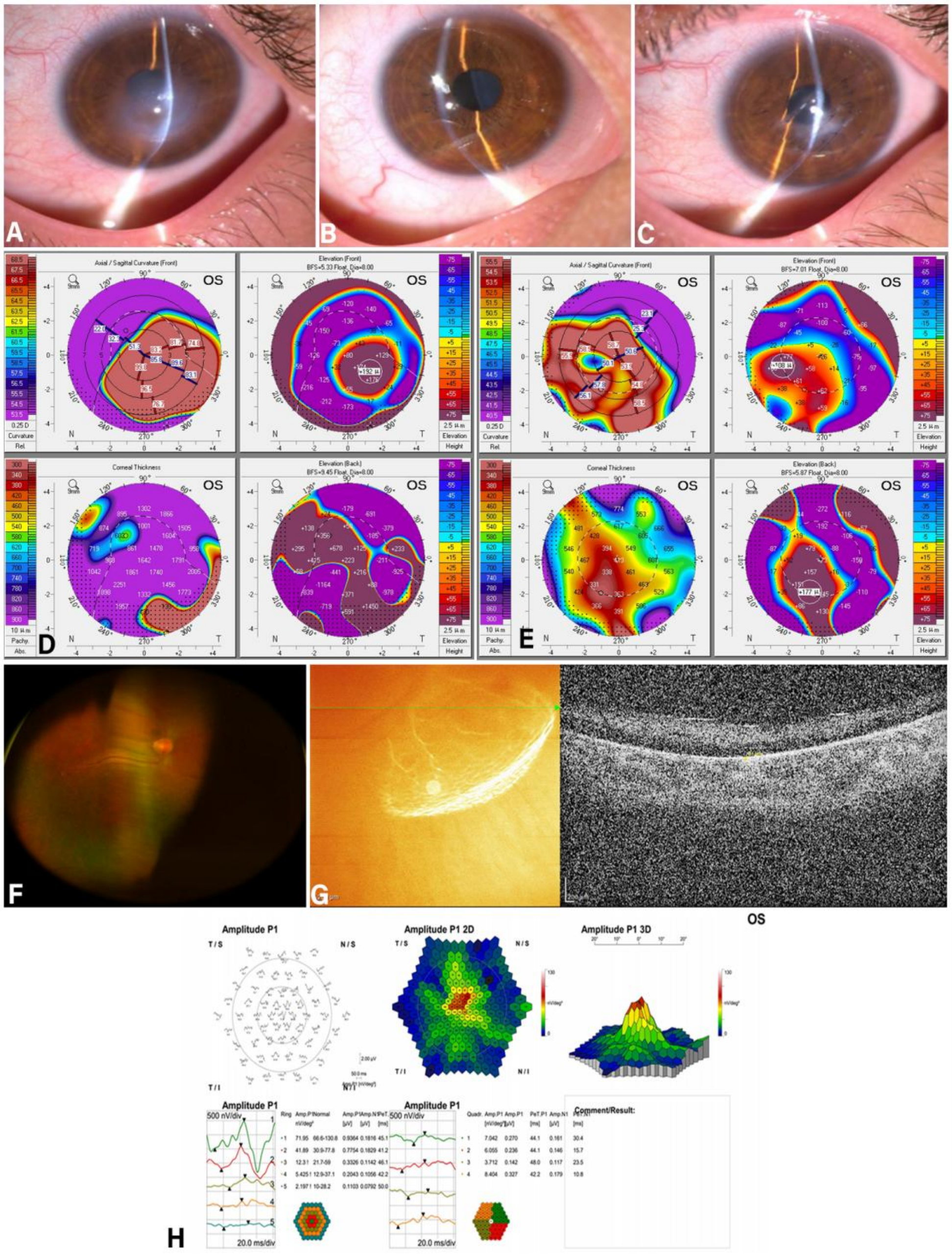

Supplementary Figure 2..

(A) Slit lamp examination show the left eye: Conjunctival hyperemia, corneal edema, with a faintly visible rupture of the central corneal posterior elastic layer.
(B) Right eye: Corneal transparency, corneal cone-shaped protrusion.
(C) Follow-up examination at 4 months postoperatively revealed mild conjunctival hyperemia in the left eye, resolution of corneal edema, suture fixation without displacement, and residual patchy opacities in the central cornea.

(D) Corneal topography shows the left eye with a maximum K-value of 102.0D, thinnest point thickness of 571μm, anterior surface height of 192μm, and posterior surface height of 415μm. The right eye exhibits a maximum K-value of 81.1D, thinnest point thickness of 101μm, anterior surface height of 202μm, and posterior surface height of 224μm.

(E) Four months post-surgery, corneal topography revealed a maximum K-value of 63.2D in the left eye, with a thinnest point thickness of 306 μm, anterior surface height of 108 μm, and posterior surface height of 177 μm.

(F) Fundus photography reveals diffuse osteoclast-like pigment deposition in the peripheral retina of the right eye.

(G) Macular OCT reveals disappearance of the retinal ellipsoid zone and atrophy of the retinal pigment epithelium (RPE) layer.

(H) Electroretinogram findings: Central 1-2 rings show mild reduction in amplitude density, rings 3-5 show severe reduction, and the 3D spike shows moderate reduction; all quadrants in the quadrant diagram show severe reduction in amplitude density.

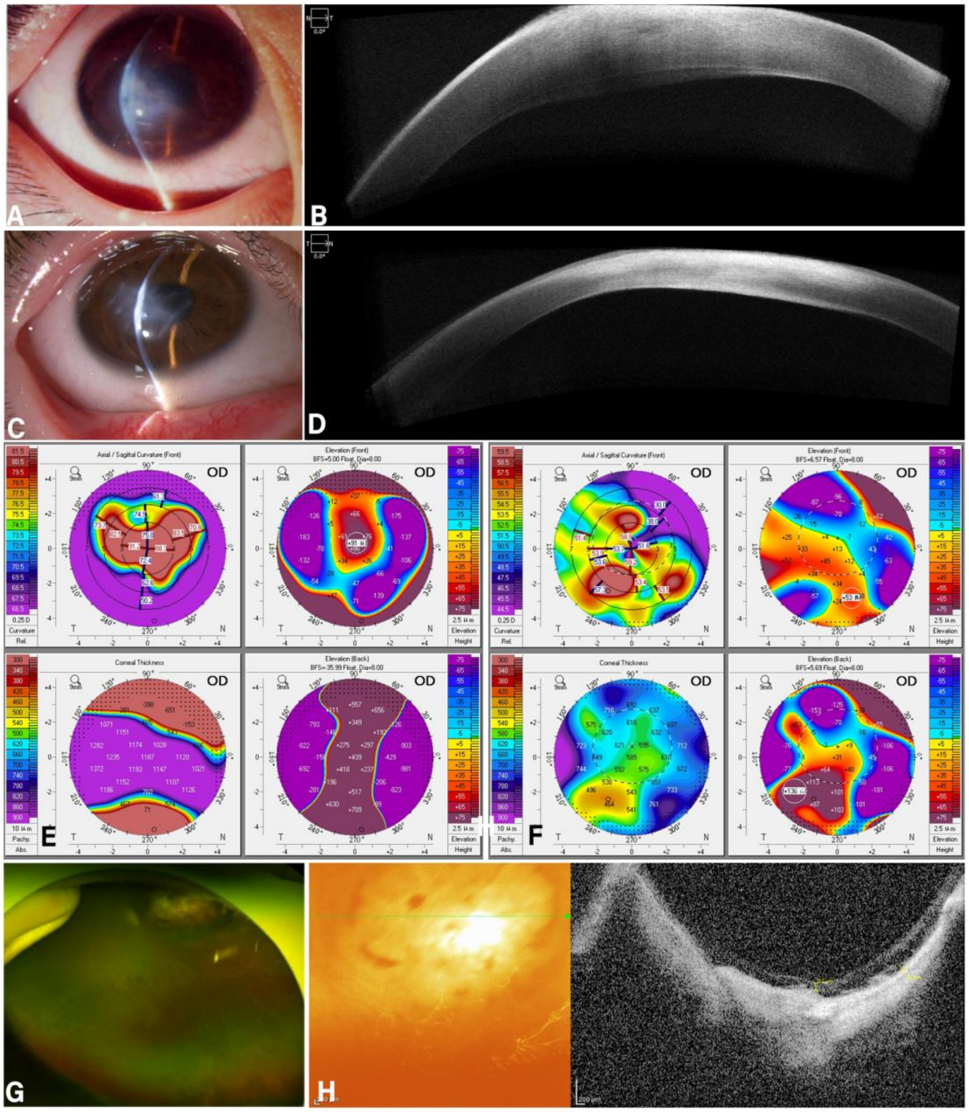

Supplementary Figure 3.

(A)Preoperative examination :Slit-lamp examination indicate corneal edema in the right eye.

(B)Anterior segment OCT shows corneal hydrops with stromal clefts.

(C,D)Three months after surgery, corneal edema in the right eye has completely resolved.

(E)The corneal topography demonstrates features consistent with keratoconus: Right eye maximum K-value: 100.4D Corneal edema thickness: 1372μm Anterior surface height: 91μm Posterior surface height: 439μm.

(F)Corneal topography: Right eye maximum K-value 67.3D, corneal edema thickness 451μm, anterior surface height 53μm, posterior surface height 136μm.

(G)Fundus photography reveals a waxy yellow disc in both eyes, with macular retinal atrophy accompanied by pigment encirclement.

(H)Macular OCT reveals flattening of the foveal center and atrophy of the retinal pigment epithelium layer.

2.2 Supplementary Table

Changes in the cornea after combination of deep lamellar corneal suturing and anterior chamber gas injection in patients with acute corneal edema

	**before the intervention**					**after the intervention**				
	Kmax,D	TCT, µm	ACE ,µm	PCE, µm	CCT, µm	Kmax,D	TCT, µm	ACE, µm	PCE, µm	CCT, µm
**Case1**	83.0	24	87	962	1498	42.7	506	73	476	523
**Case2**	102	571	192	415		63.2	306	108	117	
**Case3**	100.4	71	91	439	814	67.3	451	53	136	504

Kmax:Maximum keratometry value;TCT:Thinnest corneal thickness;ACE:Anterior corneal elevation; PCE:Posterior corneal elevation;CCT: Central corneal thickness

The central corneal thickness of case 2 was not collected
